# Tagasaste, leucaena and paulownia: three industrial crops for energy and hemicelluloses production

**DOI:** 10.1186/s13068-021-01930-0

**Published:** 2021-04-08

**Authors:** Alberto Palma, Javier Mauricio Loaiza, Manuel J. Díaz, Juan Carlos García, Inmaculada Giráldez, Francisco López

**Affiliations:** 1 Research Center in Technology of Products and Chemical Processes, PRO2TECS-Chemical Engineering Department, “El Carmen” Campus, University of Huelva, Huelva, Spain; 2 Prof. J.C. Vílchez-Martín” Chemistry Department, El Carmen” Campus, University of Huelva, Huelva, Spain

**Keywords:** Tagasaste, Leucaena, Paulownia, Biomass combustion, Hemicellulose extraction

## Abstract

**Background:**

Burning fast-growing trees for energy production can be an effective alternative to coal combustion. Thus, lignocellulosic material, which can be used to obtain chemicals with a high added value, is highly abundant, easily renewed and usually inexpensive. In this work, hemicellulose extraction by acid hydrolysis of plant biomass from three different crops (*Chamaecytisus proliferus, Leucaena diversifolia* and Paulownia trihybrid) was modelled and the resulting solid residues were used for energy production.

**Results:**

The influence of the nature of the lignocellulosic raw material and the operating conditions used to extract the hemicellulose fraction on the heat capacity and activation energy of the subsequent combustion process was examined.

The heat power and the activation energy of the combustion process were found to depend markedly on the hemicellulose content of the raw material. Thus, a low content in hemicelluloses resulted in a lower increased energy yield after acid hydrolysis stage.

The process was also influenced by the operating conditions of the acid hydrolysis treatment, which increased the gross calorific value (GCV) of the solid residue by 0.6–9.7% relative to the starting material. In addition, the activation energy of combustion of the acid hydrolysis residues from *Chamaecytisus proliferus* (Tagasaste) and Paulownia trihybrid (Paulownia) was considerably lower than that for the starting materials, the difference increasing with increasing degree of conversion as well as with increasing temperature and acid concentration in the acid hydrolysis.

The activation energy of combustion of the solid residues from acid hydrolysis of tagasaste and paulownia decreased markedly with increasing degree of conversion, and also with increasing temperature and acid concentration in the acid hydrolysis treatment. No similar trend was observed in *Leucaena diversifolia* (Leucaena) owing to its low content in hemicelluloses.

**Conclusions:**

Acid hydrolysis of tagasaste, leucaena and paulownia provided a valorizable liquor containing a large amount of hemicelluloses and a solid residue with an increased heat power amenable to efficient valorization by combustion. There are many potential applications of the hemicelluloses-rich and lignin-rich fraction, for example as multi-components of bio-based feedstocks for 3D printing, for energy and other value-added chemicals. 
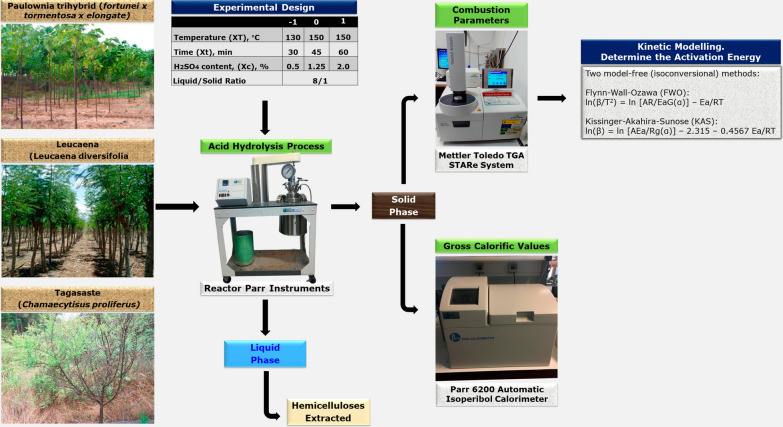

## Background

Economic and social development in recent years has raised a number of problems on environmental resources [1, 2] and fostered a search for new types of energy to replace conventional fossil fuels with cleaner, more sustainable alternatives [3, 4]. Industries worldwide are increasingly obtaining renewable energy from biomass [5] while strengthening environmental protection. In fact, plant biomass has proved an excellent source of renewable energy. This is particularly so with energy crops that are not only highly productive but also efficient sinks for CO_2_ or at least “neutral” as regards carbon emissions to the atmosphere [3, 6, 7].

European countries are increasingly concerned with sustainable, environmentally friendly production of energy, which is increasingly being obtained from renewable sources. Using wood for energy production has the added advantage that it can promote forest culturing in countries with a low forest area and help improve post-harvesting management practices in young forests [8–10].

Burning fast-growing trees for energy production can be an effective alternative to coal combustion [3, 11]. Thus, lignocellulosic material, which can be used to obtain chemicals with a high added value [12–14], is highly abundant, easily renewed and usually inexpensive. At present, fast-growing biomass provides 44–65% of all renewable energy used in European countries and reduces greenhouse gas emissions by 209 million tons each year [15, 16].

In this work, we assessed the potential of three different plant species (viz., *Chamaecytisus proliferus*, *Leucaena diversifolia* and *Paulownia fortunei x tormentosa x elongata*) for energy production*.* These species have been evaluated in other works for their biomass production and edaphoclimatic adaptability by the same and other authors. Therefore, the main goal of the article is the evaluation and comparison of these three energy crops or industrial crops from two important points of view: energy and chemical products from hemicelluloses *Chamaecytisus proliferus,* popularly known as tagasaste, is a fast-growing bushy legume typically used as forage but can also be cropped for energy production [17–21]. Tagasaste is highly productive, with more than 18 t ha^–1^ years^–1^ in some cases. In addition, it provides sheltering from the wind, helps control erosion and soil salinity, fertilizes soil and contains enough protein for feeding to animals [22–25]. *Leucaena diversifolia* (leucaena) is a legume growing up to 6–20 m under Mediterranean conditions with a high biomass productivity: usually more than 50 t ha^–1^ years^–1^ [26], or even up to 70 ton ha^–1^ years^–1^ after 7 years [27]. This species has a high re-sprouting ability [28] and provides not only wood and high-quality forage, but also energy and useful biorefinery products [27, 29]. *Paulownia fortunei x tormentosa x elongata* (Paulownia trihybrid) is a native plant from China, where it has been known and grown for 2600 years. Paulownia is a fast-growing species with an average fibre length of 1.42 mm and low water requirements. By virtue of its high productivity and low density, paulownia trees can provide 1 m^3^ of wood after 5–7 years of growth [30, 31]. Paulownia wood is used to construct cabinets, musical instruments, mouldings, furniture and veneers, and also to obtain cellulose [32, 33].

In addition, the transition to bio-economy and sustainable development requires not only reducing our dependence on fossil fuels and mitigating greenhouse gas emissions [34, 35], but also developing the technology needed for more extensive valorization of lignocellulosic biomass from energy crops [36, 37].

In this work, we examined the extraction of hemicelluloses by acid hydrolysis for their subsequent combustion. The starting acid hydrolysis was that the hydrolysis residue would have an increased heat power and combustion efficiency relative to the original material, and also that the resulting liquor would contain large amounts of hemicelluloses and other valorizable derivatives.

The primary aim of this work was to optimize the extraction of hemicelluloses from biomass of three different energy crops (viz., *Chamaecytisus proliferus*, *Leucaena diversifolia* and *Paulownia fortunei x tormentosa x elongata*) and optimize energy production by combustion. For this purpose, we used the solid residue from the acid hydrolysis of the three raw materials for combustion in combination with a factorial experimental design and multiple regression polynomial models to examine the influence of the nature of the lignocellulosic material and the operating conditions of the acid hydrolysis treatment on the heat power of the solid residue and the activation energy of the combustion process.

## Results and discussion

### Acid hydrolysis

As noted earlier, the primary aim of this work was to optimize energy production by combustion of three different lignocellulosic materials (viz., tagasaste, leucaena and paulownia trihybrid) previously subjected to hemicellulose extraction by acid hydrolysis. Obviously, removing some material (mainly hemicelluloses fraction) should be expected to decrease the amount of energy to be obtained; however, the hemicellulose-rich acid hydrolysis liquor obtained should be valorizable and combustion of the resulting solid residue more energy-efficient by effect of the acid hydrolysis pretreatment reducing its activation energy. These hypotheses were tested by characterizing the raw materials, the solid residues obtained at different points of the proposed experimental design and the results of the thermogravimetric analysis. There are many potential applications of the hemicelluloses-rich and lignin-rich fraction, for example as multi-components of bio-based feedstocks for 3D printing [38], for energy [39] and other value-added chemicals [40].

Table 1 shows the results of the characterization of the three raw materials and various others studied by Alfaro et al. [18] and Feria et al. [27], as well as those for *Eucaliptus globulus*, which was used as reference. All chemical determinations were made in triplicate and the coefficient of variation was less than 5% in all cases. As can be seen, the xylan content of leucaena was nearly 20% lower than were those of tagasaste and paulownia trihybrid, and the total hemicellulose content was almost 22% lower. On the other hand, the content in mineral ash of leucaena was 75% higher than that of tagasaste, but the calorific power of the former was lower than were those of the other two raw materials. As shown below, the differences in composition resulted in marked differences in energy yield among the raw materials.

**Table 1:** Chemical composition and Gross Calorific Value (GCV) of the three raw materials

Property^1^	Tagasaste		Leucaena		Paulownia trihybrid		*Eucalyptus globulus*
	This work	Other authors^2^	This work	Other authors^3^	This work	Other authors^4^	Other authors^5^
Ash, %	0.8	0.7 ± 0.1	1.4	1.4 ± 0.1	n.d	0.9 ± 0.1	0.5 ± 0.1
Ethanol extract., %	2.8	2.3 ± 0.1	n.d	1.7 ± 1.1	n.d	4.6 ± 0.1	3.1 ± 0.8
Glucan, %	41.4	38.9 ± 3.4	32.2	38.0 ± 2.4	34.2	44.0 ± 3.3	44.4 ± 3.7
Lignin, Klason %	20.1	19.8 ± 1.9	21.5	24.8 ± 0.6	27.2	27.8 ± 1.1	27.6 ± 4.9
Xylan, %	18.5	19.9 ± 1.3	15.5	15.7 ± 0.1	18.3	15.7 ± 0.2	18.8 ± 1.4
Araban, %	0.6	0.6 ± 0.3	1.0	1.5 ± 0.3	1.1	1.1 ± 0.1	2.5 ± 1.1
Acetylgroups, %	3.3	4.4 ± 0.6	2.1	3.3 ± 0.5	3.3	4.4 ± 0.2	3.4 ± 0.1
GCV, kJ/kg o.d.b	19,375	19,592 ± 28	19,060	18,981 ± 102	19,362	20,300	19 326 ± 160

n.d. not determined

^1^All percentages are referred to dry matter

^2^[18, 19]

^3^[27, 78, 79]

^4^[30, 31, 80]

^5^[81, 82]

Table 2 shows the extraction yield, the hemicelluloses extracted (in respect hemicelluloses fraction in raw material), and Gross/Inferior calorific values of the solid residues from acid hydrolysis of the three target raw materials. As can be seen, hemicelluloses were more efficiently extracted from paulownia and tagasaste than they were from leucaena, which is consistent with the decreased content of the last species in this fraction. The calorific power of the solid residue exceeded that of the raw material in the three species. This result is consistent with increased dissolution of the hemicellulose fraction, which was that with the lowest calorific power [41].

**Table 2:** Experimental design, extraction yield, amount of hemicellulose extracted and calorimetric value of the solid residues from acid hydrolysis of the three raw materials

*X*_*C*_	*X*_*T*_	Tagasaste		Leucaena		Paulownia trihybrid	
		Extraction yield/Hemicelluloses extracted (%)	GCV at constant volume (J/g o.d.b.)	Extraction yield/Hemicelluloses extracted (%)	GCV at constant volume (J/g o.d.b.)	Extraction yield/Hemicelluloses extracted (%)	GCV at constant volume (J/g o.d.b.)
1	1	52.8/87.1	20,889	68.9/79.4	19,869	45.9/95.3	21,222
1	− 1	48.9/79.1	19,619	84.3/42.9	19,353	45.3/87.1	20,400
− 1	1	64.0/82.2	20,714	58.8/75.4	19,292	44.8/77.2	20,673
− 1	− 1	83.3/40.2	19,506	71.7/52.9	18,916	65.1/38.2	19,642
1	0	53.7/71.5	19,905	77.2/54.7	19,149	47.5/92.3	20,713
− 1	0	76.5/50.2	19,724	65.6/56.1	19,038	56.0/41.4	20,112
0	1	61.1/86.5	20,492	59.4/77.7	19,873	51.9/60.2	20,784
0	− 1	68.1/63.6	19,443	73.7/47.8	19,265	59.1/60.1	19,753
0	0	70.2/62.0	19,605	66.2/55.2	19,361	53.2/57.2	20,165
0	0	69.9/62.0	19,604	66.3/54.8	19,305	54.1/57.5	20,200

*GCV* Gross Calorific Value

The data in Table 2 were used to model the relationship between the previous properties and the conditions of the acid hydrolysis treatment. For this purpose, the data were subjected to multiple regression as described in “Acid hydrolysis” section. The equations of the models thus obtained are shown in Table 3 together with their statistics (adjusted *R*^2^ and Snedecor’s *F*-value). The values used to establish the equations were the average of 3 measurements each. The differences between the experimental values and those estimated from the previous equations were less than 5% in all instances; in addition, *F*-values exceeded 51 and *R*^2^ was greater than 0.96 in all cases.

**Table 3:** Equations for the dependent variables (gross calorific value, extraction yield and amount of hemicelluloses extracted) as a function of the independent variables (acid concentration and temperature)

Equations		*R*^2^	Snedecor’s *F*-value
Tagasaste			
(1)	GCV = 19 603.0 + 77.8 *X*_*C*_ + 587.5 *X*_*T*_ + 212.9 *X*_*C*_*X*_*C*_ + 365.9 *X*_*T*_*X*_*T*_	0.99	812
(2)	Yield = 69.3 − 11.4 *X*_*C*_ − 3.7 *X*_*T*_ − 3.5 *X*_*C*_*X*_*C*_ − 3.9 *X*_*T*_*X*_*T*_ + 5.8 *X*_*C*_*X*_*T*_	0.99	211
(3)	HE = 62.5 + 10.8 *X*_*C*_ + 12.2 *X*_*T*_ − 2.1 *X*_*C*_*X*_*C*_ + 12.0 *X*_*T*_*X*_*T*_ − 8.5 *X*_*C*_*X*_*T*_	0.99	325
Leucaena			
(4)	GCV = 19 315.5 + 82.8 *X*_*C*_ + 274.8 *X*_*T*_ + 218.7 *X*_*T*_*X*_*T*_	0.97	115
(5)	Yield = 66.4 + 5.7 *X*_*C*_ − 7.1 *X*_*T*_ + 4.7 *X*_*C*_*X*_*C*_ − 0.6 *X*_*C*_*X*_*T*_	0.99	1460
(6)	EH = 55.2 − 1.2 *X*_*C*_ + 14.8 *X*_*T*_ + 7.5 *X*_*T*_*X*_*T*_ + 3.5 *X*_*C*_*X*_*T*_	0.99	1633
Paulownia trihybrid			
(7)	GCV = 20 186.6 + 318 *X*_*C*_ + 480.6 *X*_*T*_ + 221.8 *X*_*C*_*X*_*C*_ + 77.8 *X*_*T*_*X*_*T*_ − 52.2 *X*_*C*_*X*_*T*_	0.99	325
(8)	Yield = 54.5 − 4.5 *X*_*C*_ − 4.5 *X*_*T*_ − 3.8 *X*_*C*_*X*_*C*_ + 5.2 *X*_*C*_*X*_*T*_	0.96	51
(9)	EH = 57.6 + 17.5 *X*_*C*_ + 10.8 *X*_*T*_ + 16.5 *X*_*C*_*X*_*C*_ − 7.7 *X*_*C*_*X*_*T*_	0.98	214

GCV Gross Calorific Value, HE amount of hemicelluloses extracted, *X*_*C*_ acid concentration, *X*_*T*_ temperature

The differences between the experimental values and those estimated using the equations never exceeded 5% of the former

The independent variables are expressed in coded units (Eq. 1)

The coefficients of the linear terms in the equations of Table 3 allow one to envisage the influence of the independent variables on the dependent variables. Thus, maximizing hemicellulose extraction and Gross Calorific Value would require using high levels of the two independent variables (temperature and time); on the other hand, obtaining the best possible yield would entail using low levels of the previous variables.

**Fig. 1:**
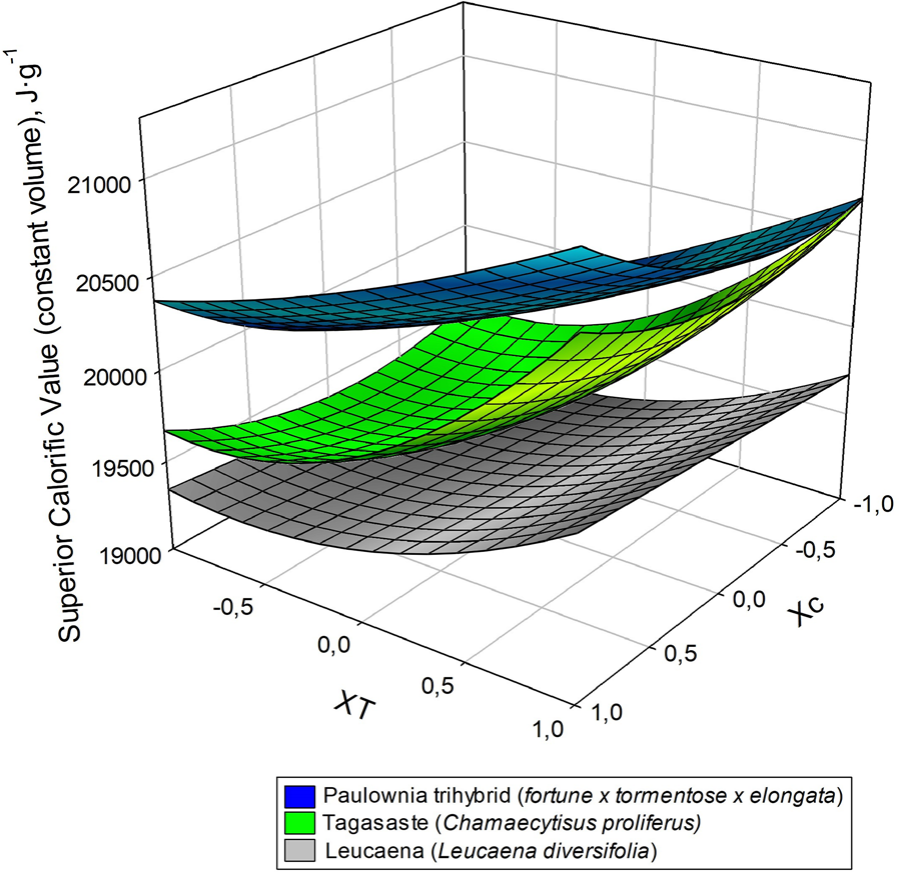
Variation of the Gross Calorific Value of the three species as a function of the acid concentration and temperature

**Fig. 2:**
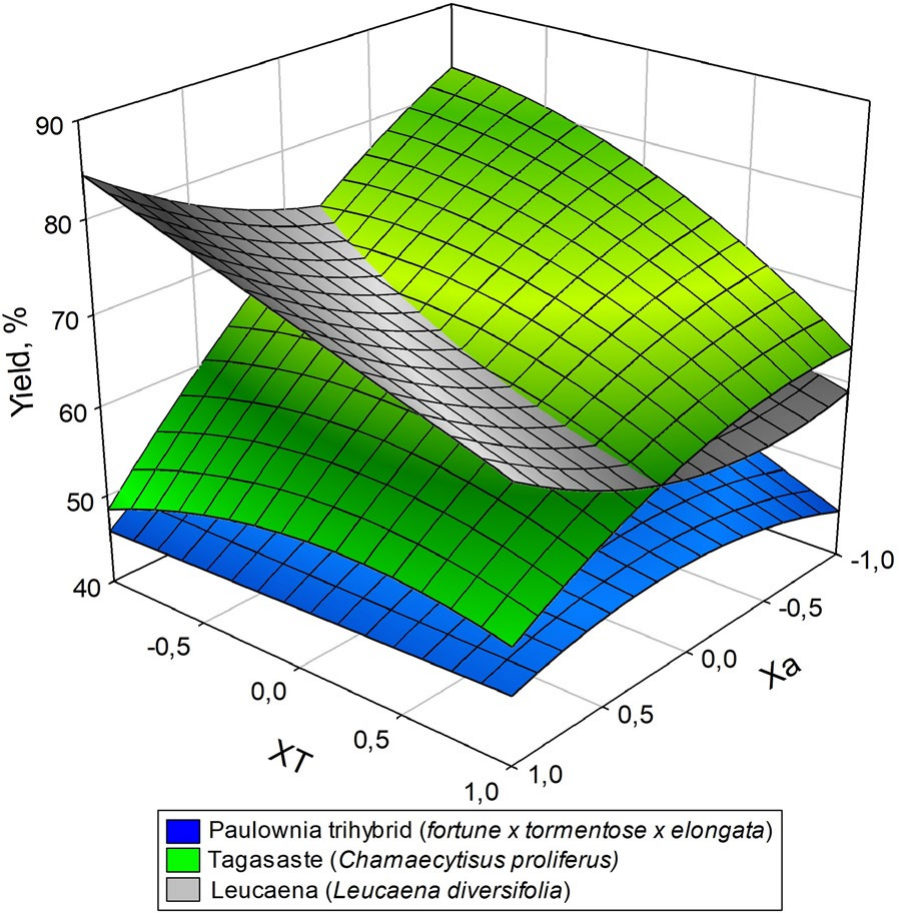
Variation of the extraction yield of the three species as a function of the acid concentration and temperature

**Fig. 3:**
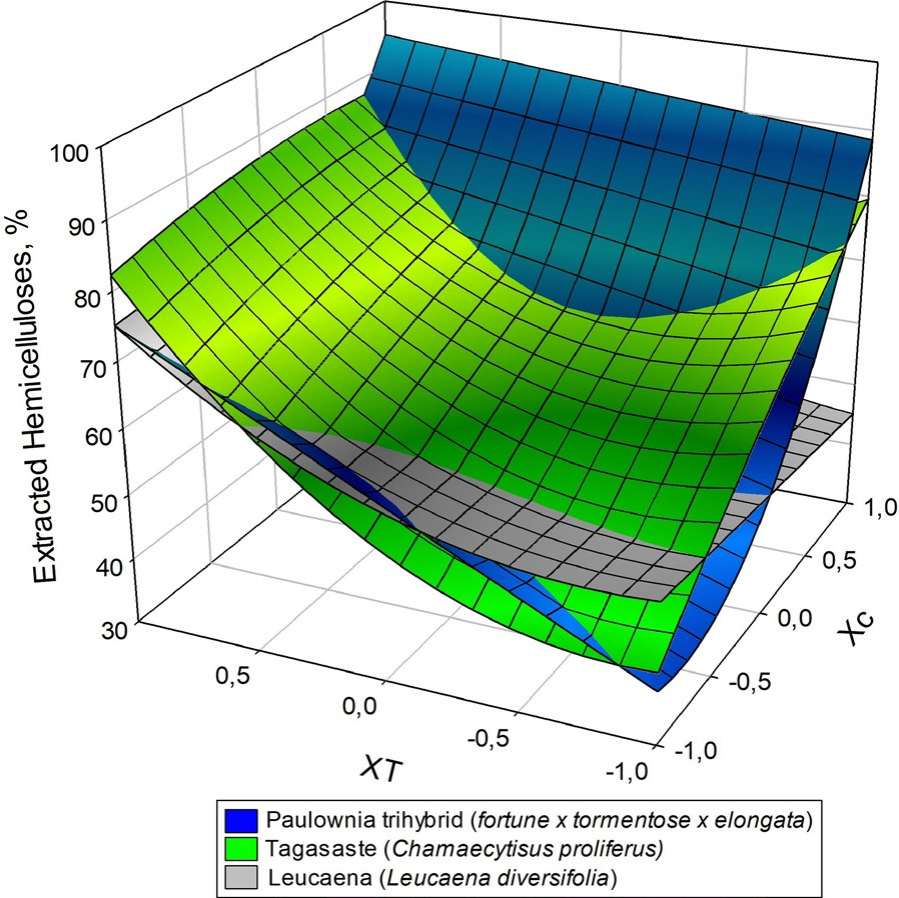
Variation of amount of hemicelluloses extracted from the three species as a function of the acid concentration and temperature

Comparing the influence of independent variables by analyzing the models in Table 3 is difficult. There are linear, quadratic, and interaction terms between variables. Therefore, it is easier to interpret the interaction between independent variables graphically. Figures 1, 2, 3 are response surfaces intended to more clearly illustrate the relationships between variables. As can be seen in Fig. 1, the calorific power of the solid residue from acid hydrolysis of leucaena was considerably lower than were those of the other two raw materials. This, however, cannot have been the sole result of leucaena containing less hemicelluloses. In fact, paulownia and tagasaste had a similar calorific power despite their substantial differences in hemicellulose content. Seemingly, extracting hemicelluloses by acid hydrolysis of the raw material had an additional, marked effect on the calorific power of the solid residue from paulownia and tagasaste. This is also apparent from Figs. 2 (yield) and 3 (hemicellulose extraction), where the response surfaces illustrate the differential influence of acid hydrolysis on the three raw materials. The patterns of extracted hemicelluloses for tagasaste and leucaena are similar (see Fig. 3). For extraction yield, the patterns are significatively differents only a high alkali concentration. Probably due to the higher extract content and polysaccharide content in tagasaste than in leucaena.

Although the calorific power of a solid biofuel such as lignocellulosic biomass obviously differs among raw materials, it may also differ depending on how a particular material is treated prior to combustion. In theory, acid hydrolysis of lignocellulosic biomass should cause the most easily hydrolysed fractions (hemicelluloses here) to be removed in the acid hydrolysis liquor, thereby increasing the calorific power of the resulting solid residue. In previous paragraph the high correlation between GCV and hemicelluloses extracted was established. In fact, the cellulose and lignin fractions are known to have a higher calorific power than the hemicellulose fraction [42, 43]. This was indeed the case with the three raw materials studied. Thus, based on Eqs. 3, 6 and 9 in Table 3, the Gross Calorific Value (GCV) for the solid residues from acid hydrolysis under extreme operating conditions of tagasaste, leucaena and paulownia was 0.7–7.6%, 0.6–4.4% and 1.4–9.7% higher than that for the respective raw material. As can be seen from Fig. 1, the increase in GCV for paulownia exceeded those of the other two materials throughout the operating range. This was partly the result of raw, untreated paulownia having an increased GCV relative to the other two species (see Table 1) [18, 27].

Because an increased GCV can result from increased extraction of hemicelluloses, and hence from an increase in the proportions of cellulose and lignin in the acid hydrolysis solid residue, we examined the variation of the degree of hemicellulose extraction with the acid concentration and temperature used in the acid hydrolysis treatment preceding combustion. As can be seen in Fig. 3, hemicellulose extraction from tagasaste, leucaena and paulownia was 40.9, 52.6 and 37.6%, respectively, at experimental point (− 1 − 1—see Table 2), and increased to 86.9, 79.8 and 94.7%, respectively at (+ 1, + 1). The increase in GCV was thus consistent with that in hemicellulose extraction from the three raw materials. GCV and hemicellulose extraction were greatest for paulownia, followed by tagasaste and leucaena, the last species being the least likely to benefit from acid hydrolysis in terms of GCV.

The change in total yield included the effect of acid hydrolysis on the cellulose and polyphenol fractions. As can be seen from Fig. 2, paulownia exhibited the greatest decrease in extraction yield (from 65.1 to 44.8%). This result is consistent with previous comments on hemicellulose extraction and GCV increase. However, as can also be seen from the response surfaces for tagasaste and leucaena in Fig. 2, the raw materials responded rather differently to acid hydrolysis. Thus, the results for leucaena were much more strongly dependent on temperature (especially at high acid concentrations), whereas those for tagasaste were more markedly dependent on the acid concentration. This can be observed in the figures and Eqs. 2, 5 and 8 in Table 3. The lineal term coefficient for temperature is higher for leucaena. In addition, the interaction term between temperature and acid concentration is negative for leucaena (see Eq. 5).

**Fig. 4:**
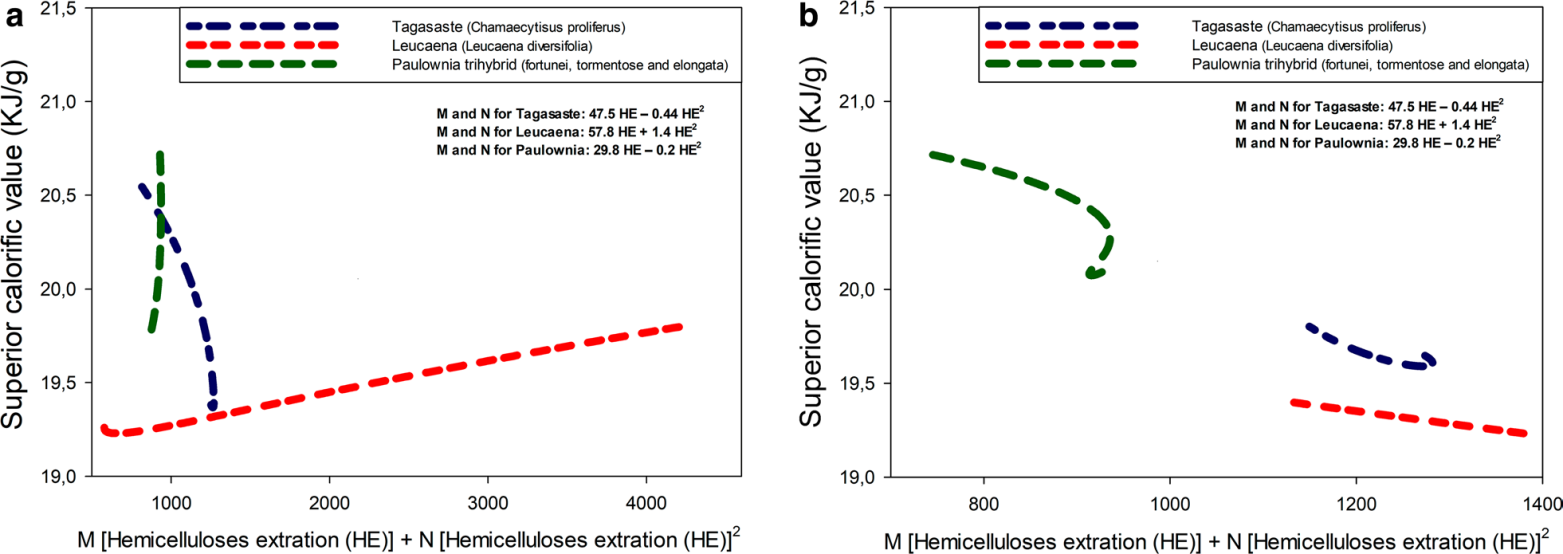
Correlation between the Gross Calorific Value and hemicellulose extraction from the three species at a constant temperature of 150 °C (**a**) and a constant acid concentration of 1.25% (**b**)

The dependence of the GCV increase on the degree of hemicellulose extraction was examined using various correlations between the two variables in Eqs. 1, 3, 4, 6, 7 and 9 of Table 3. Using multiple regression models to assess the dependence of GCV on the independent variables revealed that the quadratic and interaction terms in the equations, in combination with the hemicellulose extraction rate in linear, quadratic and cubic form as an added independent variable, allowed very robust fitting with models, where the two independent variables of the process, hemicellulose extraction and their quadratic terms were all statistically significant. Thus, adjusted-*R*^2^ values were in the region of 0.99 and *F* values all greater than 400. Although none of the models is shown, the coefficients for the terms “hemicellulose extraction” (HE) and “hemicellulose extraction squared” (HE^2^) were used to plot the variation of GCV with the following linear combination of the two terms: 47.58·HE–0.4415·HE^2^ for tagasaste,–57.88·HE + 1.46·HE^2^ for leucaena and 29.85·HE–0.23811·HE^2^ for paulownia. As can be seen from Fig. 4, which shows the results for the three raw materials at the central values of the independent variables, the two types of variables were highly correlated, albeit with differences among the three species.

### Thermogravimetric analysis of the combustion process

As stated above, extracting hemicelluloses by acid hydrolysis alters the GCV of a raw material but can also influence the activation energy of the resulting solid residue. In fact, the lignocellulosic biomass pyrolysis has a complex mechanism studied by several authors, for example George et al. [44]. In this work, we hypothesized that using appropriate acid hydrolysis conditions would provide solid residues subsequent combustion of which would occur with a lower activation energy than in the starting material, thereby increasing the combustion efficiency [45, 46]. This would be an additional benefit to the obtainment of a potentially valorizable hemicellulose-rich liquor and a solid residue with a greater calorific power than the original material.

**Fig. 5:**
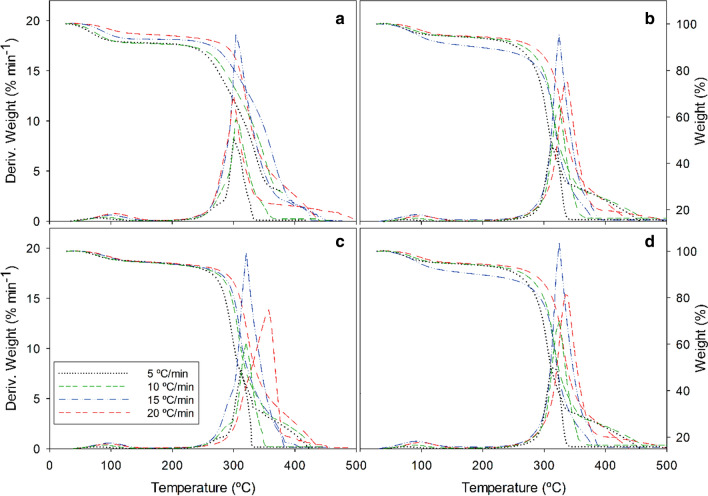
TGA and DTG curves for tagasaste combustion at a variable heating rate (5, 10, 15 or 20 °C min^−1^). Raw material (**a**) and solid residue from hydrolysis with 2% H_2_SO_4_ at 170 °C for 60 min (**b**), 1.25% H_2_SO_4_ at 150 °C for 45 min (**c**) and 0.5% H_2_SO_4_ at 130 °C for 30 min (**d**)

**Fig. 6:**
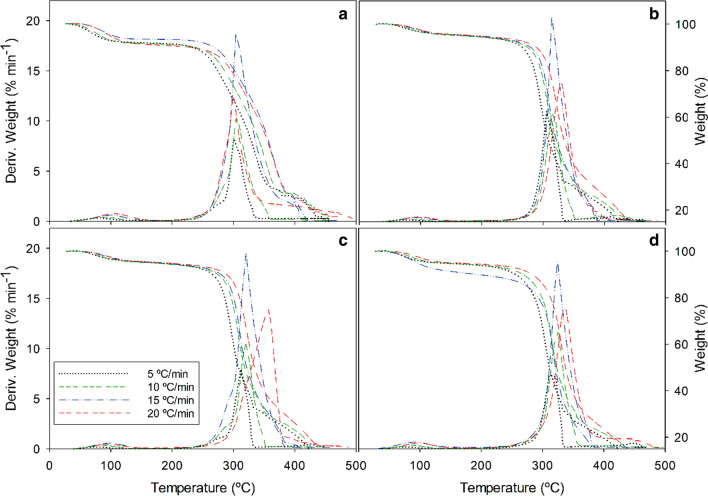
TGA and DTG curves for paulownia trihybrid combustion at a variable heating rate (5, 10, 15 or 20 ºC min^−1^). Raw material (**a**) and solid residue from hydrolysis with 2% H_2_SO_4_ at 170 °C for 60 min (**b**), 1.25% H_2_SO_4_ at 150 °C for 45 min (**c**) and 0.5% H_2_SO_4_ at 130 °C for 30 min (**d**)

The activation energy of combustion of the acid hydrolysis solid residue from the three raw materials was determined by thermogravimetric analysis as described in “Kinetic analysis”section. Tests were conducted at the central and extreme values of the experimental, design, namely: (− 1, − 1), (0, 0) and (+ 1, + 1). Figures 5, 6 illustrate the variation of the mass loss (TGA) and differential mass loss (DTG) as a function of temperature and the heating rate (5, 10, 15 or 20 °C min^–1^) for each raw material (data not shown for leucaena). The thermograms correspond to the solid residues obtained at the central and extreme points of the experimental design for the acid hydrolysis treatment. Each material exhibited the typical four TGA regions and DTG peaks corresponding to mass losses by evaporation and sequential combustion of the hemicellulosic, cellulosic and polyphenolic fraction (lignin). Specifically, the first DTG peak, at 60–120 °C, corresponds to the removal of intrinsic and absorbed water. That for the thermal decomposition of polysaccharides usually appears from 200 to 300 °C in hemicellulose and 350–450 °C cellulose. Our peaks for the two mass losses were largely overlapped in the region from 200 to 400 °C. Alternatively, a small shoulder corresponding to the hemicellulosic fraction was observed, consistent with the results for other materials [47, 48]. The peaks for the cellulosic fraction shifted to higher temperatures as the heating rate (ºC min^−1^) was increased from 5 to 20 °C min^−1^.

The polyphenolic (lignin) fraction is a complex combination of benzene–propane units spanning a broad range of molecular weights and including very large, heavily reticulated structures [49, 50], that are thus very thermally stable [51]. In this complex situation, the polyphenolic fraction decomposes thermally over a broad temperature range and gives no characteristic peaks [52]. However, our materials gave sharp peak above 400 °C that differed markedly depending on the heating rate and the conditions of the acid hydrolysis treatment. Thus, thermal decomposition started at a lower temperature (130 °C) in the raw materials than in the solid residues from the hydrolysis of paulownia (130 °C) and tagasaste (150 °C). In leucaena, however, the degradation peaks for the polyphenolic fraction appeared in the region of 400 °C in all cases. Finally, the degradation peaks for the thermal degradation of lignin in the DTG curves became increasingly broad as the heating rate (ºC min^−1^) was raised.

### Kinetic analysis

As noted in the previous section, the activation energy of combustion of the solid residue from acid hydrolysis of each raw material was determined by TGA and DTG analysis in combination with the Kissinger–Akahira–Sunose (KAS) and Flynn–Wall–Ozawa (FWO) methods. Therefore, as pointed out in “TGA and kinetic modelling” section, the kinetic parameters (Ea and A) of both the initial solid residues and those obtained after subsequent acid hydrolysis, through the slopes of the mass loss versus heating rate (ºC min^−1^) plots, can be calculated. The graphs corresponding to the selected methods (KAS and FWO) showed high-level adjustment lines. These statistical values support the accuracy of the proposed models.

**Fig. 7:**
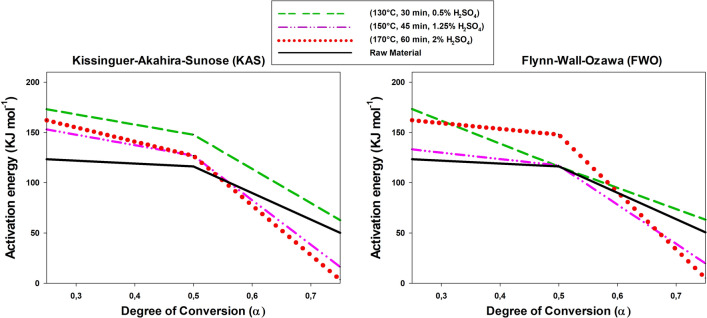
Variation of the activation energy of combustion of tagasaste as a function of the degree of conversion

**Fig. 8:**
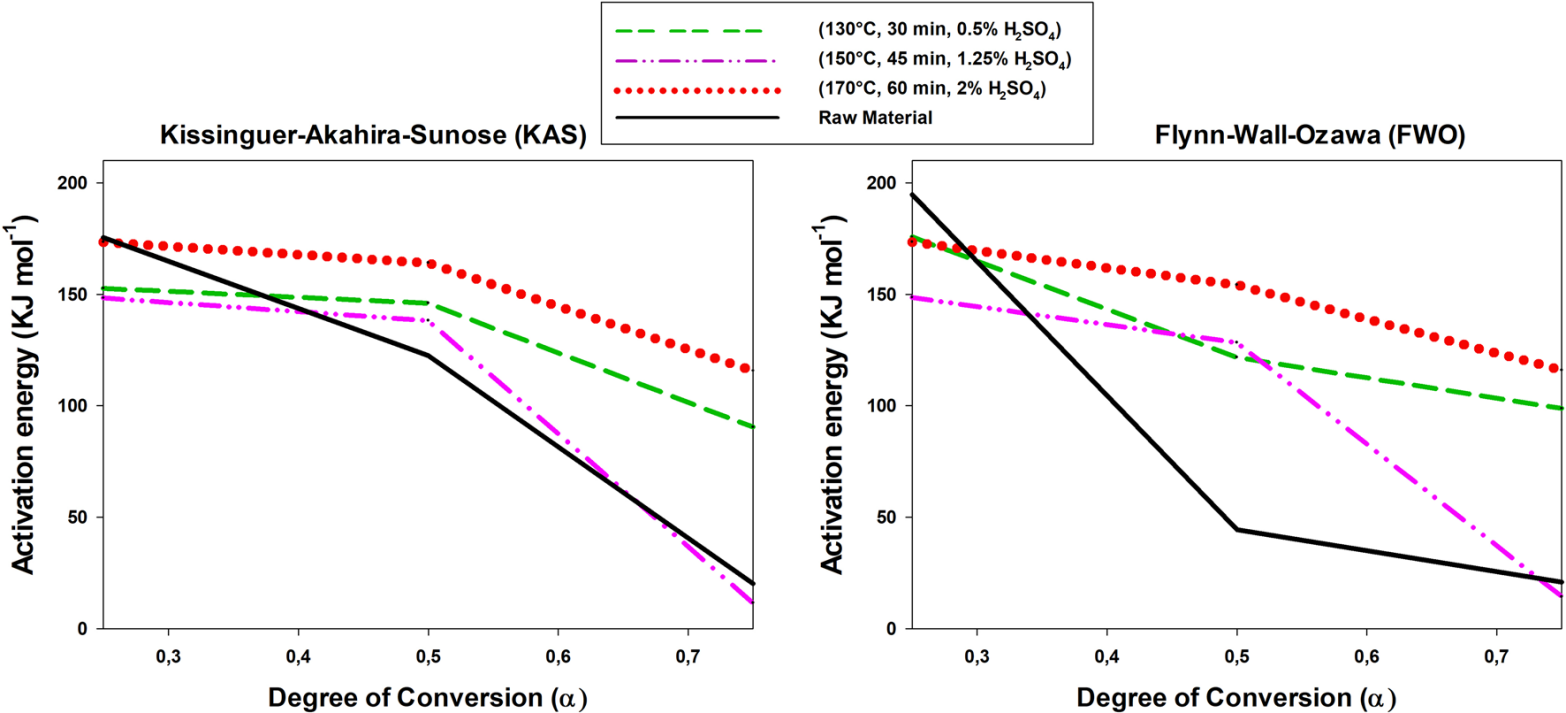
Variation of the activation energy of combustion of leucaena as a function of the degree of conversion

**Fig. 9:**
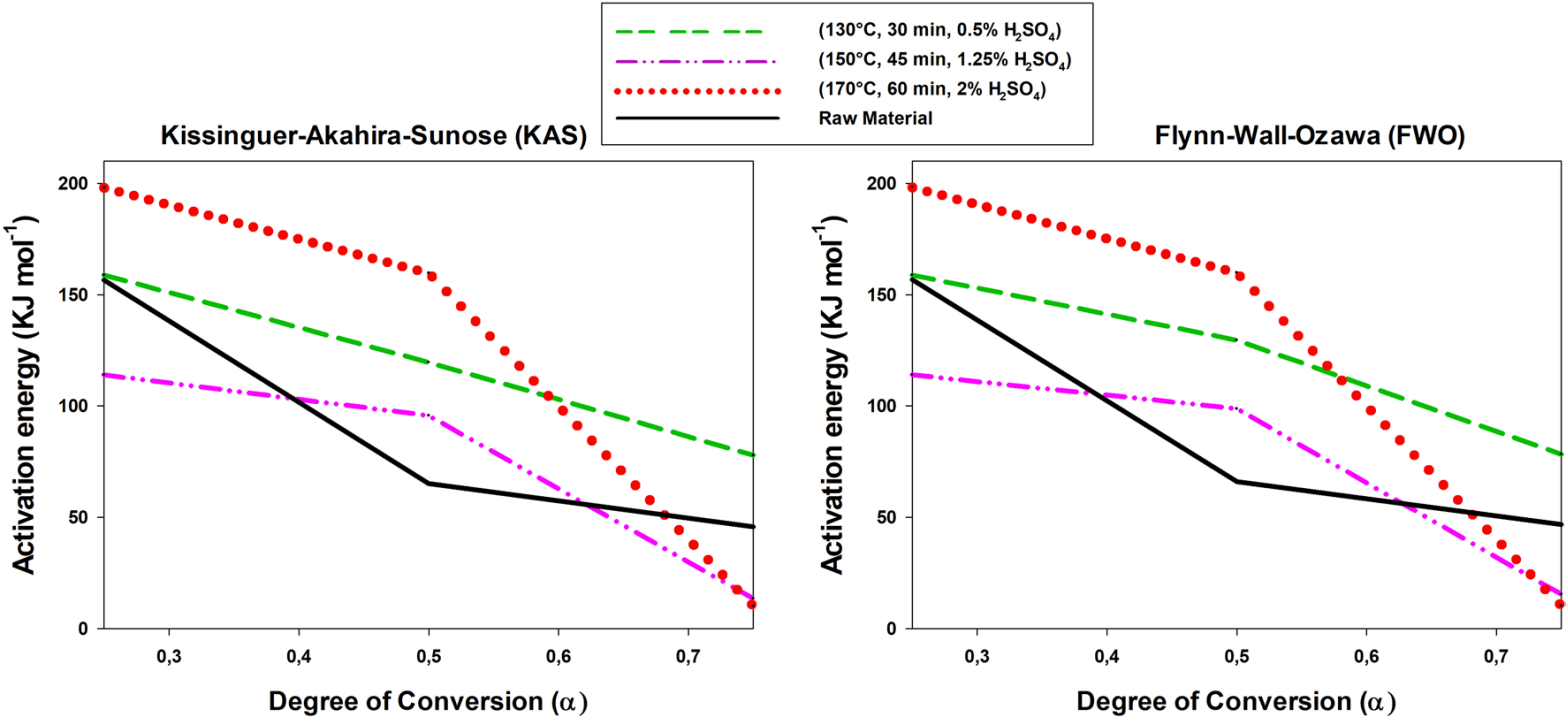
Variation of the activation energy of combustion of paulownia trihybrid as a function of the degree of conversion

Figures 7, 8, 9 show the variation of the activation energy as a function of the degree of conversion in the combustion of the solid residues from acid hydrolysis of the raw materials at three different points in the experimental design. Overall, the results are similar to those previously reported by other authors [53, 54]; in addition, they differed little between the two methods and the differences arose from their using different parameters. In any case, the results were quite good and consistent with those of Kok et al. [55] and Parthasarathy et al. [56]. As can be seen, combustion of the solid residue was a typical stepwise reaction, where the activation energy decreases with increasing degree of conversion, this was particularly sow at *α* > 0.50.

In theory, because the raw materials had a higher hemicellulose content than the solid residues obtained from their acid hydrolysis, the former should require a lower activation energy (*E*_a_) for combustion at low degrees of conversion [57]. In fact, the cellulosic and polyphenolic (lignin) fractions have increased *E*_a_ values [54, 58]. This was indeed the case with tagasaste here, but, strictly, neither with paulownia nor with leucaena, the *E*_a_ values at low degrees of conversion for the last species fell above those for all other combustion processes at the central and extreme points of the experimental design for the acid hydrolysis process. The differences were consistent with the decreased contents in xylan and acetyl groups of leucaena relative to the other two raw materials and, also possibly, of the increased mineral content of this species. In fact, acid hydrolysis removed minerals to a greater extent from leucaena than it did from tagasaste or paulownia, which may have led to a decreased activation energy of combustion in the resulting solid residue [59].

As expected, *E*_a_ in the combustion process decreased with increasing degree of conversion [60], particularly in those solid residues obtained at increased temperatures or acid concentrations. In fact, strong acid hydrolysis conditions facilitated depolymerization of the cellulosic or polyphenolic fraction [53, 61, 62], thereby facilitating subsequent combustion. This was indeed the case with tagasaste and paulownia at degrees of conversion above 0.6 or 0.7, where the *E*_a_ values for combustion of the acid hydrolysis solid residues obtained at acid concentrations higher than 1.25%, temperatures above 150 °C and reaction times longer than 45 min were lower than those for the raw materials. On the other hand, leucaena exhibited no well-defined operating range for acid hydrolysis and provided a solid residue with a lower activation energy than the raw material itself.

## Conclusions

The results obtained in this work allow us to draw the following conclusions:

- Extracting hemicelluloses by acid hydrolysis of tagasaste, leucaena and paulownia prior to their valorization by combustion provides a hemicellulose-rich liquor and a solid residue with a higher calorific power than the raw material.
- The hemicellulose content of the raw material had a strong influence on the calorific power and activation energy of combustion of the solid residue from acid hydrolysis of the three raw materials. In fact, reducing the initial hemicellulose content increased the efficiency and energy yield of combustion of the hydrolysed material.
- The operating conditions of the acid hydrolysis process also influenced the combustion process, the Gross Calorific Value (GCV) of which was increased by 0.6–9.7% relative to the starting material. The increase in GCV upon acid hydrolysis was strongly correlated with the degree of hemicellulose extraction, albeit to a different extent depending on the particular raw material.
- The activation energy of combustion of the solid residues from acid hydrolysis of tagasaste and paulownia decreased markedly with increasing degree of conversion, and also with increasing temperature and acid concentration in the acid hydrolysis treatment. No similar trend was observed in leucaena owing to its low content in hemicelluloses.

## Methods

### Characterization and storage of the raw materials. Acid hydrolysis of wood samples

Samples of tagasaste (*Chamaecytisus proliferus*) consisting of 0.5–5.0 cm thick stems and branches were collected from Trigueros (Huelva), southwestern Spain. Leucaena (*leucaena diversifolia*) biomass was obtained from another plantation in Huelva, where plots had been kept for 7 years. The plants had been planted at 3 months of age in two plots of sandy loam soil of pH 6–8 to which no fertilizer was applied. The distance between plants was 0.6–1.8 m and their density 10,800 plants ha^–1^. A completely randomized block design with four replicates was used.

Paulownia trihybrid (*fortunei x tormentosa x elongata*) was harvested after 3 years of growth in the southwestern Spanish region of Extremadura and supplied by Vicedex Europa (Barcelona, Spain).

For the three raw materials, the harvested material being ground in a hammermill after removing leaves and non-wood portions. The size particle was the usual en paper industry: 2–3 cm in length and 0.5 cm (approximately) in wide. For this particle size not diffusional differences were appreciated. The chips were air dried until constant weight and stored in hermetic bags.

For characterization, the three raw materials were ground to a particle size less than 0.5 mm. Leucaena, tagasaste, and paulownia trihybrid were characterized chemically using TAPPI T264 cm-07 [63] for moisture content, TAPPI 211 om-02 [64] for ash content and TAPPI T204-om-07 [65] with Soxhlet extraction (95% ethanol, 5 h) for ethanol extractables. After characterization, the three raw materials were subjected to quantitative acid hydrolysis with 72% H_2_SO_4_. The resulting hydrolysates were analysed chemically according to TAPPI T249-em-09 [66]. Monomeric sugars (xylose, arabinose and glucose) and acetic acid in the acid hydrolysis liquor were determined by high performance liquid chromatography (HPLC), using an Aminex HPX-87H ion-exchange column at 30 °C as stationary phase and 0.05 M H_2_SO_4_ at a flow-rate of 6 mL min^–1^ as mobile phase. Monosaccharide contents were expressed in terms of xylan, araban and glucan. Klason lignin was determined according to TAPPI T222 om-11 [67].

### Acid hydrolysis procedure

The acid hydrolysis pretreatment used to extract hemicelluloses was performed in a 2 L stainless steel reactor from Parr Instruments Co. (Moline, IL, USA). The liquid/soil ratio was kept constant at 8 kg_water_ kg_raw material_^–1^ (o.d.b.). The independent variables of the extraction process were temperature (130, 150 or 170 °C) and acid (H_2_SO_4_) concentration (0.5, 1.25 or 2%). Based on existing recommendations [26], the reaction time was 60 min in all tests. Once extracted, the liquid fraction with hemicelluloses were separated from solid residue by filtration and washed in water, the solid being air-dried and weighed to calculate the yield of the process.

The solid residue from the acid hydrolysis treatment was characterized for yield and the amount of hemicelluloses (xylose, arabinose and acetic acid) extracted under identical conditions for the three raw materials. The residue was also characterized for calorific power according to standards CEN/TS 14918:2005 E (“Solid biofuels method for the determination of calorific value”) [68] and UNE164001 EX [69]. A Parr 6300 automatic Isoperibol calorimeter, a CGA 540 connector, 99.5% pure oxygen and a maximum pressure of 2500 psig were used for this purpose.

### Acid hydrolysis. Multiple regression models and experimental design

The acid hydrolysis process was modelled and optimized using polynomials comprising linear and quadratic terms of the process variables, and their mutual interactions, the equations thus established being fitted by multiple regression. A 2^*n*^ central composite factor design was used to reduce the number of tests needed while ensuring the absence of significant covariances between dependent variables. In this way, the dependent variables (yield, hemicelluloses extracted and Gross Calorific Value) were related to the independent variables (temperature and acid concentration). Generally these variables and operation time had interacted during acid hydrolysis but the effect of operation time, in the range of operation selected, is significantly lower than temperature and acid concentration [70]. Modelling required previously normalizing the ranges spanned by the independent variables according to Eq. 1 and statistically identifying the significant influences in the coefficients. Thus, no term with a coefficient *p* > 0.05 as per Student’s *t*-test or spanning a confidence interval of less than 95% was included:

1 $$X_{n} = \frac{{X - \overline{X} }}{{\left( {X_{{{\text{max}}}} - X_{{{\text{min}}}} } \right)/2}}$$

where *X* is the absolute value of the independent variable concerned, *X̅* is its mean value, and *X*_max_ and *X*_min_ are its maximum and minimum value, respectively.

Three levels each of independent variable were used, namely: 0.5, 1.25 and 2% H_2_SO_4_, and 130, 150 and 170 °C. The minimum number of tests needed, *N*, was calculated to be 2^*n*^ + 2·*n* + *c*, where *n* is the number of independent variables and *c* that of replicates of the central point in the experimental design (Eq. 2). *N* was, therefore, 2^2 +^ 2 × 2 + 2 = 10. The experimental results were fitted to the following second-order polynomial equation:

2 $$Y = \, a_{0} \, + \, \sum\limits_{i = 1}^{n} { \, b_{i} } X_{{{\text{ni}}}} \, + \, \sum\limits_{i = 1}^{n} { \, c_{i} } X_{{{\text{ni}}}}^{2} \, + \, \sum\limits_{i = 1, \, j = 1}^{n} { \, d_{{{\text{ij}}}} } X_{{{\text{ni}}}} X_{{{\text{nj}}}} { (}i{ < }j{)}$$

where *X* denotes independent variables and *Y* dependent variables, the coefficients *a*_*o*_, *b*_*i*_, *c*_*i*_, and *d*_ij_ being constant unknown characteristics estimated from the experimental data.

The results were assessed with the software Statistica 10.0 (StatSoft, Inc., Tulsa, OK, USA). Universal fitting statistics such as *R*^2^ and Snedecor’s *F*-value were also used. *R*^2^ > 0.85 or *F* > 5 were taken to be acceptable.

### TGA and kinetic modelling

Combustion of the solid residue obtained by acid hydrolysis of tagasaste, leucaena and paulownia trihybrid was examined using non-isothermal thermogravimetric analysis (TGA) to determine the kinetic constants for the process. Such constants were calculated from mass losses at different temperatures and times.

The thermo–chemical combustion of the solids was studied using a TGA/DSC1 STARe System thermo–gravimetric analyzer from Mettler (Toledo, OH, USA). Tests were carried out using an amount of sample of 50–100 mg, under N_2_ and O_2_ streams at 15 and 20 mL min^–1^, respectively, the temperature being raised from 25 to 500 °C at 5, 10, 15 or 20 °C min^–1^.

TGA data can be processed in different ways to estimate kinetic parameters, but are usually handled with model-free fitting techniques. In fact, determining kinetic parameters for combustion reactions by model-based fitting is a difficult task [71, 72] and better done with isoconversional methods (e.g., model-free methods such as those based on single-step kinetics). Specially, prominent among such methods are the Kissinger–Akahira–Sunose (KAS) and Flynn–Wall–Ozawa (FWO) methods. Both KAS and FWO are integral isoconversional methods. Activation energy, without assumption for reaction type, could be calculated using of points with the same conversion from the measurements with different heating rates. KAS method analysis show graphics Log (Heating rate /T2) versus inverse temperatures and FWO method analysis shows graphic Log (Heating rate) versus inverse temperatures both for the points with the same conversion as the straight line of each conversion value. In addition, Activation Energy and Pre-exponential factor for this conversion values are found from the slope an intersect of these lines.

Among the main advantages for both methods are that could be used for multiple-step reactions and each reaction point could be evaluated and could be used under dynamic and isothermal measurements. However, for parallel and independent simultaneous reactions gives the mean Ea values. The FWO method, which is the more commonly used [58, 73], describes reaction changes as a function of temperature as follows [74, 75]:

3 $$\ln \left( \beta \right){\text{ = ln }}\left( {\frac{AEa}{{Rg\;\left( \alpha \right)}}} \right) \, - { 2}{\text{.315 }} - { 0}{\text{.4567}}\frac{Ea}{{RT}}$$

where *A* is the pre-exponential factor, *β* the heating rate, *E*_a_ the activation energy, *g* a conversion function, *T* temperature and *R* the gas constant. Plotting the logarithm of the heating rate (ln *β*) against the reciprocal temperature (1/*T*) at a given degree of conversion (*α*) and different heating rates provides a linear graph.

The KAS method is a modified version of the Arrhenius equation [76, 77], requiring no prior knowledge of the exact thermal degradation value. Rather, this method integrates Eq. (4) from the initial condition (*a* = 0 at *T* = *T*_0_) to obtain Eq. (5):

4 $$\left( {\frac{{{\text{d}}\alpha }}{{{\text{dT}}}}} \right) = \frac{A}{\beta } \cdot {\text{exp}}\left( {\frac{{ - {\text{Ea}}}}{{{\text{RT}}}}} \right) \cdot f{(}\alpha {)}$$

5 $$G{(}\alpha {)} = \int_{0}^{\alpha } {\frac{d\alpha }{{f{(}\alpha {)}}} = \frac{{\text{A}}}{\beta }} \, \int_{{T_{0} }}^{T} {{\text{exp}}\left( {\frac{{ - {\text{Ea}}}}{{{\text{RT}}}}} \right) \cdot {\text{dT}}}$$

The KAS method is based on the Coats-Redfern approximation:

6 $$G{(}\alpha {)} = \frac{A}{\beta }\frac{{{\text{RT}}^{2} }}{{{\text{Ea}}}}{\text{exp}}\left( {\frac{{ - {\text{Ea}}}}{{{\text{RT}}}}} \right)$$

rearrangement of which and conversion into natural logarithmic form yields

7 $${\text{ln }}\left( {\frac{\beta }{{T^{2} }}} \right) = \ln \left( {\frac{{{\text{AR}}}}{{{\text{EaG(}}\alpha {)}}}} \right) - \frac{{{\text{Ea}}}}{{{\text{RT}}}}$$

The activation energy can be calculated from thermogravimetric curves obtained at a constant degree of conversion and variable heating rates. The TGA curves for the three raw materials were similar, which is consistent with their also similar composition (cellulosic, hemicellulosic and polyphenolic polymers).

## Data Availability

All data generated or analyzed during this study are included in this published article.

## Abbreviations

GCV: Gross Calorific Value
HE: Hemicellulose extraction
HE^2^: Hemicellulose extraction squared
TGA: Variation of the mass loss
DTG: Variation of the differential mass loss
KAS: Kissinger–Akahira–Sunose method
FWO: Flynn–Wall–Ozawa method
Ea: Activation energy
